# Contact with recovered peers: buffering disempowering service experiences and promoting personal recovery in serious mental illness

**DOI:** 10.1192/bjo.2019.72

**Published:** 2019-11-08

**Authors:** Bronte McLeod, Denny Meyer, Greg Murray, Fiona Foley, Nev Jones, Neil Thomas

**Affiliations:** Centre for Mental Health, Swinburne University of Technology, Australia; Professor of Statistics, Centre for Mental Health, Swinburne University of Technology, Australia; Professor of Psychology, Centre for Mental Health, Swinburne University of Technology, Australia; Project Manager, Centre for Mental Health, Swinburne University of Technology, Australia; Assistant Professor, Department of Psychiatry, University of South Florida, USA; Associate Professor of Psychology, Centre for Mental Health, Swinburne University of Technology; and The Alfred Hospital, Australia

**Keywords:** Peer contact, involuntary treatment, internalised stigma, personal recovery, serious mental illness

## Abstract

**Background:**

Mental health patients can experience involuntary treatment as disempowering and stigmatising, and contact with recovered peers is cited as important for countering stigma and fostering agency and autonomy integral to recovery.

**Aims:**

To advance understanding of the interaction between involuntary treatment and contact with recovered peers, and explore hypothesised relationships to mechanisms of self-evaluation relevant to recovery.

**Method:**

Eighty-nine adults diagnosed with serious mental illness completed items to assess involuntary treatment experience and the extent of prior contact with recovered peers, the Internalised Stigma of Mental Illness Scale, the Self-efficacy for Personal Recovery Scale, the Questionnaire about the Process of Recovery and relevant demographic and clinical scales.

**Results:**

Contact with recovered peers was found to moderate the effects of involuntary treatment on internalised stigma. Sequential conditional process models (i.e. moderated mediation) then demonstrated that conditional internalised stigma (i.e. moderated by contact with recovered peers) mediated the indirect effect of involuntary treatment on recovery-specific self-efficacy, which in turn influenced recovery. Compared with those with low contact with recovered peers, recovery scores were 3.54 points higher for those with high contact.

**Conclusions:**

Although study methods limit causative conclusions, findings are consistent with proposals that contact with recovered peers may be helpful for this patient group, and suggest this may be particularly relevant for those with involuntary treatment experience. Directions for future research, to further clarify measurement and conceptual tensions relating to the study of (dis)empowering experiences in mental health services, are discussed in detail.

Recent decades have seen significant changes in mental health service delivery, with the recovery paradigm progressively instantiated in policies and guidelines, particularly in English-speaking countries of the Global North.^1–3^ Mental health services for people diagnosed with serious and persisting mental health problems increasingly prioritise personal recovery, which has been defined as ‘living a satisfying, hopeful and contributing life even with limitations caused by the illness’.^4^ From a research viewpoint, there has been burgeoning interest in systems, services and treatment regimens for factors that may facilitate or impede personal recovery.^5,6^ Two findings are particularly relevant to the present project.

First, personal recovery may be especially relevant to those with involuntary treatment experiences, such as forced treatment in hospital or mandated community treatment. A number of studies have found these experiences can be detrimental to the integrity of the self and impede agency and autonomy, as well as reinforcing prevailing, negative mental illness stereotypes.^7–11^ Second, there is evidence that one important facilitator of recovery may be contact with peers with shared mental health-related experiences who are further along the recovery pathway.^7,12,13^

Reported benefits of contact with recovered peers include countering the impact of stigma and discrimination by challenging stereotypes, and engendering self-efficacy beliefs by offering individuals a ‘road map for how to navigate their recovery journeys’ through vicarious experiential learning.^7,8,14^ Internalised stigma, the process of endorsing, internalising and applying mental illness stereotypes to oneself,^15^ may be an important common variable in understanding how both involuntary treatment experiences and contact with recovered peers influence personal recovery. Moreover, multiple studies have shown that internalising stigma acts as a barrier to recovery by undermining a view of oneself as capable and agentic,^16^ with lower levels of internalised stigma predicting greater self-efficacy and recovery.^15,17,18^

These findings suggest there is a complex interplay between disempowering (involuntary treatment) and empowering (contact with recovered peers) experiences, and self-evaluative processes in the prediction of recovery among people with persisting mental health problems. However, the field is challenged by a lack of measurement and conceptual clarity regarding these constructs and their interrelationships.

## The present study

**Fig. 1:**
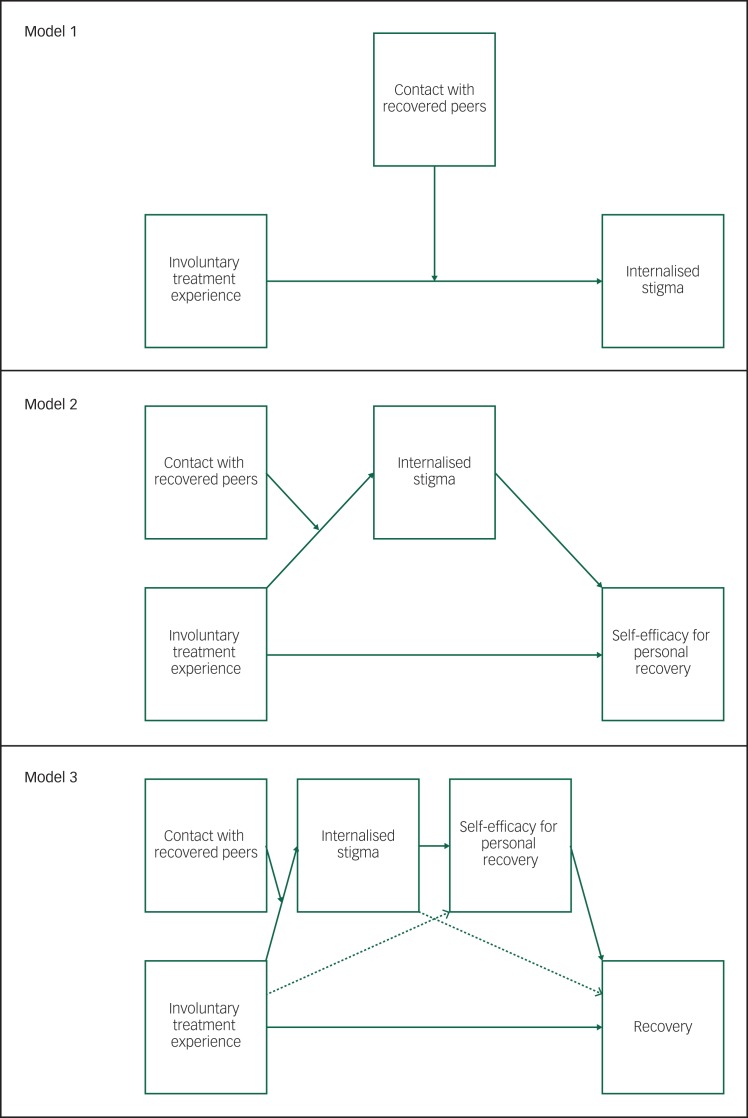
Conceptual models illustrating hypothesised conditional direct and indirect effects for contact with recovered peers, involuntary treatment, internalised stigma, recovery-specific self-efficacy and recovery.

In order to advance the scientific study of patient experiences in mental health services, the aim of the present study was to systematically examine the interactive relationship between involuntary treatment experience and contact with recovered peers, and the intrapersonal mediating processes that may help to explain their impact on personal recovery. We hypothesised that: (a) contact with recovered peers moderates the effect of involuntary treatment on internalised stigma (hypothesis 1); (b) conditional internalised stigma (i.e. moderated by contact with recovered peers) mediates the indirect effect of involuntary treatment on recovery-specific self-efficacy (hypothesis 2); and (c) self-efficacy mediates the conditional indirect effect of internalised stigma on recovery (hypothesis 3). The full complexity of this moderated multiple-mediation model was tested using conditional process analysis (i.e. ordinary least-squares regression-based path analysis) and our predictions are captured in the conceptual models in Fig. 1. In the hypothesised (conditional) models, only the effect of involuntary treatment on internalised stigma is moderated by contact with recovered peers. Further explication of the theoretical and empirical rationale for the present study is available in the extended introduction provided as supplementary File 1 available at https://doi.org/10.1192/bjo.2019.72.

In the hypothesised (conditional) models, only the effect of involuntary treatment on internalised stigma is moderated by contact with recovered peers.

## Method

### Context

Data for this study were collected as part of the Self-Management and Recovery Technology (SMART) research programme in Victoria, Australia.^19^ Data for the current study were drawn from baseline data from one component of the SMART research programme (SMART-Therapy).

### Participants

The participants comprised 89 adults aged 18–63 (mean 41.24, s.d. = 11.04; *n* = 46, 51.7% men), recruited from clinical and community mental health services via referral from practitioners, print and online advertisements and publicity within services. Inclusion criteria were: (a) aged between 18 and 65 years inclusive; (b) diagnosis of non-organic psychotic disorder (schizophrenia-related disorder or bipolar disorder or major depressive disorder with psychotic features present within the past 2 years), confirmed using the Structured Clinical Interview for DSM-IV-TR Axis I Disorders (SCID);^20^ (c) sufficient conversational English for meaningful participation; (d) overall intellectual functioning within normal limits (having an IQ greater than 70, as estimated by the Wechsler Test of Adult Reading^21^). Exclusion criteria were: (a) initiation of a new antipsychotic medication, or commencement or completion of a formal psychological treatment within the previous 8 weeks; (b) in-patient admission within the previous 8 weeks. Additional demographic data are presented in supplementary Table 1.

### Procedure

This study was performed in accordance with the Declaration of Helsinki and was approved by Human Research Ethics Committees at The Alfred (study number 139–14), St Vincent's Hospital Melbourne (study number 041.14), Melbourne Health (study number 2014.087), Austin Health (HREC/15/Austin/308; study number ND15/308) covering Peninsula Health (SSA/15/PH/49) and Eastern Health (SERP09-2016), Swinburne University (study number 2014/119) and Deakin University (study number 2014-285). All participants provided full informed consent prior to commencement. Participants completed self-report measures assessing relevant study variables. The SCID and interview schedule were administered by trained research interviewers.

### Measures

Participants completed a survey containing a number of self-report scales, clinical scales and demographic questions (i.e., gender, age, ethnicity, relationship/employment status).

#### Involuntary treatment experience

Participants were asked if they had ever received involuntary treatment in a hospital or community setting. Responses informed creation of a dichotomous variable (0, no; 1, yes) reflecting lifetime experience of any involuntary treatment.

#### Contact with recovered peers

Prior contact with recovered peers was measured using a purpose-built vignette-based question, codesigned with individuals using mental health services and workers during the development phase of the SMART research programme. Participants read a brief description of a person experiencing a positive recovery from psychosis (i.e. living a personally satisfying life even though they may experience ongoing symptoms or periods of needing treatment). They then rated their extent of contact (through any medium) with people fitting this description, as a proportion of all their contact with people with persisting mental illness, on a 4-point ordinal scale (‘none at all’; ‘a few people, but they make up a very small proportion of the people with mental health problems I have encountered’; ‘a number of people, who make up a significant proportion of the people with mental health problems I have encountered’; ‘this applies to most people I have encountered’). For analyses this was dichotomised as a low (the majority of contact with people with serious mental health problems has been with people not in recovery) versus high (the majority of contact with people with serious mental health problems has been with people in recovery) variable (0, low; 1, high). Participants reported understanding this measure when administered.

#### Internalised Stigma of Mental Illness Scale (ISMIS)

The ISMIS^22^ is a 29-item scale measuring the subjective experience of stigma and discrimination. Participants rated the extent of agreement with each statement on a 4-point Likert scale ranging from 1 (strongly disagree) to 5 (strongly agree). The ISMIS contains five subscales measuring alienation (for example ‘Having a mental illness has spoiled my life’), stereotype endorsement (for example ‘People with mental illness cannot live a good, rewarding life’), perceived discrimination (for example ‘People discriminate against me because I have a mental illness’), social withdrawal (for example ‘I don't socialise as much as I used to because my mental illness might make me look or behave “weird”’) and stigma resistance (for example ‘In general, I am able to live life the way I want to’; reverse-coded subscale). Internal consistency in this study was very high (α = 0.90).

#### Self-efficacy for Personal Recovery Scale (SEPRS)

The SEPRS^23^ is a 14-item self-report scale measuring confidence in one's own ability to engage in specific behaviours relating to personal recovery from serious mental illness. Two introductory items broadly reflect self-efficacy for personal recovery (for example ‘How confident are you that in the future you will be able to live a satisfying life alongside any mental health problems you may have?’) and self-management (for example ‘How confident are you that you can do things to manage any future mental health difficulties?’), with 12 subsequent items targeting domains highlighted as important in recovery (connectedness, identity, meaning, empowerment; for example ‘Develop a view of myself beyond being a psychiatric patient’).^24^ Items are rated on a continuous scale from 0 (‘not confident I can do this at all’) to 100 (‘highly confident I can do this’) and averaged to produce an overall SEPRS score (0–100). Internal consistency in the current study was very high (α = 0.94).

#### The Questionnaire about the Process of Recovery (QPR)

The QPR^25^ is a 22-item self-report questionnaire measuring intra- and interpersonal aspects of personal recovery, which was developed in collaboration with individuals with experience of psychosis. Items are scored using a 5-point Likert scale ranging from 0 (disagree strongly) to 4 (agree strongly). Internal consistency in the current study was very high (α = 0.93).

#### Positive and Negative Syndrome Scale (PANSS)

The PANSS^26^ is a standardised interview-based measure of psychosis symptomatology, with 30 items rated on a 7-point severity scale. Interrater reliability on the PANSS was established at *r* = 0.89 between research assistants conducting interviews. Scores were dichotomised to reflect low (≤66) and high (≥67) PANSS symptom severity. Symptom severity was included as an *a priori* covariate in the present study.^27,28^

#### Data analytic strategy

Preliminary bivariate analyses were conducted to test for intelligible relationships between predictor variables, and to investigate possible confounds with categorical (age, gender, marital and employment status) and dimensional (illness duration, diagnosis, clinical and/or community service exposure, psychiatric hospital admissions and symptom severity) variables. Hypothesised moderation and conditional process models (moderated mediation) were tested using the PROCESS macro (http://www.afhayes.com) developed by Hayes^29^ for SPSS. This approach enables examination of direct and indirect effects of an independent variable on a dependent variable via one or more mediators, as well as examination of variables moderating these relationships and the inclusion of covariates.

Three progressive models (Fig. 1) were constructed to examine whether: contact with recovered peers moderates the effect of involuntary treatment experience on internalised stigma (hypothesis 1); conditional internalised stigma mediates the indirect effect of involuntary treatment on self-efficacy (hypothesis 2); and self-efficacy mediates the conditional indirect effect of internalised stigma on recovery (hypothesis 3) (these models correspond to models 1, 7 and 83 in Hayes^29^). Given the sequential nature of the models, and possible variation in relevant covariates between models, subsequent models were only tested if statistically significant support was established for the preceding model.^30^ Significant conditional direct effects of involuntary treatment on internalised stigma (hypothesis 1) were estimated at low and high levels of contact with recovered peers. Bias-corrected bootstrap confidence intervals were also generated for conditional indirect effects at both levels of contact with recovered peers based on 5000 bootstrap samples (hypotheses 2 and 3). Point estimates were considered significant if the 95% confidence intervals did not contain zero. This bootstrapping approach is considered the most effective when examining conditional process models with small sample sizes, and less vulnerable to type 1 errors, when compared with structural equation modelling.^29,31,32^ Index scores provided the difference between conditional indirect effect sizes for low and high contact with recovered peers.

## Results

### Preliminary analyses

See Table 1 for descriptive statistics and intercorrelations for all study variables. Involuntary treatment and contact with recovered peers were negatively correlated, internalised stigma was inversely correlated with self-efficacy and recovery, and self-efficacy and recovery were positively correlated. Mean scores for internalised stigma significantly differed according to high and low PANSS symptom severity (high: mean 75.71, s.d. = 14.39; low: mean 64.57, s.d. = 13.34), *t*(87) = 3.65, *P*<0.001. Lifetime experience of admission to a psychiatric hospital also significantly differed according to high and low PANSS symptom severity (yes: mean 67.22, s.d. = 13.68; no: mean 82.86, s.d. = 18.81), *t*(87) = −2.82, *P* = 0.006. Additionally, a Pearson chi-square test showed a statistically significant association between contact with recovered peers and lifetime experience of admission to a psychiatric hospital, *χ*^*2*^(1) = 4.46, *P* = 0.035. These associations can be reasonably expected, given that higher symptom severity is likely to attract greater levels of stigma and discrimination and voluntary admissions require disclosure. Symptom severity and history of psychiatric admission were therefore included as covariates in the subsequent analyses.^27,28^

**Table 1:** Summary of intercorrelations, means and standard deviations for involuntary treatment, contact with recovered peers, internalised stigma, self-efficacy for personal recovery and recovery^a^

	1	2	3	4	5
1. Involuntary treatment	–				
2. Contact with recovered peers	−0.224*	–			
3. Internalised stigma (ISMIS)	−0.040	−0.123	–		
4. Self-efficacy for personal recovery (SEPRS)	−0.050	0.060	−0.454**	–	
5. Recovery (QPR)	0.043	0.123	−0.308**	0.649**	–
Mean	0.54	0.37	68.45	65.04	56.71
s.d.	0.50	0.49	14.64	17.39	13.85

ISMIS, Internalised Stigma of Mental Illness Scale; SEPRS, Self-efficacy for Personal Recovery Scale; QPR, Questionnaire about the Process of Recovery.

a. Involuntary treatment; (0, no; 1, yes); contact with recovered peers (0, low; 1, high).

*P<0.05. **P<0.01.

### Model 1: conditional direct effect, internalised stigma

**Fig. 2:**
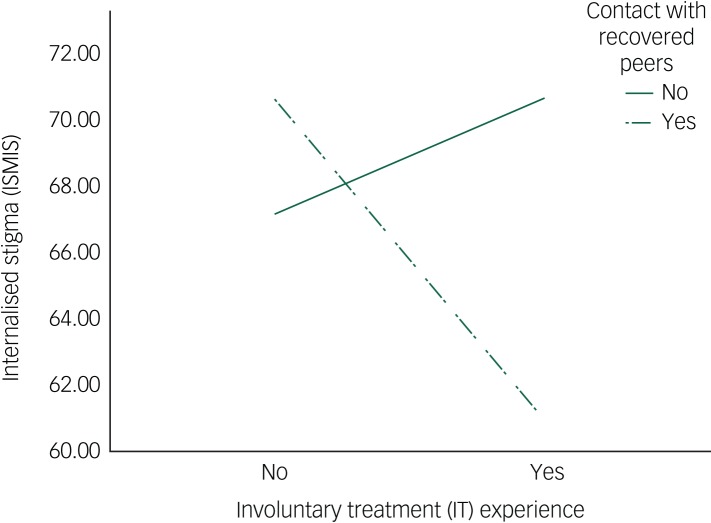
Interaction effects on internalised stigma: contact with recovered peers significantly moderated the relationship between involuntary treatment experience and internalised stigma.

ISMIS, Internalised Stigma of Mental Illness Scale; IT, involuntary treatment.

Results from model 1 were consistent with our prediction that contact with recovered peers significantly moderated associations between involuntary treatment experience and internalised stigma, *β* = −13.26 (s.e. = 5.84), *P* = 0.026 (Table 2). The interaction term explained a significant increase in the overall variance in internalised stigma, Δ*R*^2^ = 0.043, *F*(1,83) = 5.16, *P* = 0.026. Analysis of the conditional direct effects indicated that for those participants who had experienced involuntary treatment, internalised stigma was significantly lower for those with high contact with recovered peers, *β* = −9.76 (s.e. = 4.52), *t*(83) = −2.16, *P* = 0.034, 95% CI −18.75–0.77 (Table 3). When participants had experienced low contact, no significant relationship between involuntary treatment experience and internalised stigma was observed. Figure 2 further illustrates this effect.

**Table 2:** Conditional process models^a^

Antecedent	Consequent											
	Internalised stigma				Self-efficacy				Recovery			
	*β*	s.e.	*t*	*P*	*β*	s.e.	*t*	*P*	*β*	s.e.	*t*	*P*
Model 1												
IT	−1.42	3.05	−0.47	0.643								
CRP	−3.65	2.92	−1.25	0.216								
IT × CRP (interaction term)	−13.26	5.84	−2.27	0.026								
Constant	67.59	7.77	8.70	<0.001								
Symptom severity	−12.47	2.91	−4.28	<0.001								
Psychiatric hospital admission	19.23	5.5	3.49	<0.001								
Model 2												
IT	−1.42	3.05	−0.47	0.643	−2.75	3.70	−0.74	0.459				
CRP	−3.65	2.92	−1.25	0.216	–	–	–	–				
IT × CRP (interaction term)	−13.26	5.84	−2.27	0.026	–	–	–	–				
Internalised stigma	–	–	–	–	−0.53	0.14	−3.91	<0.001				
Constant	67.59	7.77	8.70	<0.001	104.06	13.32	7.81	<0.001				
Symptom severity	−12.47	2.91	−4.28	<0.001	−1.84	4.08	−0.45	0.653				
Psychiatric hospital admission	19.23	5.5	3.49	<0.001	0.15	7.10	0.02	0.983				
Model 3												
IT	−1.42	3.05	−0.47	0.643	−2.75	3.7	−0.74	0.459	0.75	2.31	0.32	0.747
CRP	−3.65	2.92	−1.25	0.216	–	–	–	–	–	–	–	–
IT × CRP (interaction term)	−13.26	5.84	−2.27	0.026	–	–	–	–	–	–	–	–
Internalised stigma	–	–	–	–	−0.53	0.14	−3.91	<.001	−0.02	0.09	−0.26	0.798
Self-efficacy	–	–	–	–	–	–	–	–	0.51	0.07	7.44	<0.001
Constant	67.59	7.77	8.70	<0.001	104.06	13.32	7.81	<0.001	33.17	10.91	3.04	0.003
Symptom severity	−12.47	2.91	−4.28	<0.001	−1.84	4.08	−0.45	0.653	2.12	2.54	0.83	0.407
Psychiatric hospital admission	19.23	5.50	3.49	<0.001	0.15	7.10	0.02	0.983	−10.43	4.43	−2.36	0.021

IT, involuntary treatment; CRP, contact with recovered peers.

a. *n* = 89. Unstandardised regression coefficients are reported. IT and CRP were mean-centred prior to analysis. In this model, only the effect of involuntary treatment on internalised stigma is moderated by contact with recovered peers. Bootstrap sample size, 5000.

**Table 3:** Model 3 summary^a^

Conditional effects analyses	IT ➙ ISMIS					IT ➙ ISMIS ➙ SEPRS					IT ➙ ISMIS ➙ SEPRS ➙ QPR				
	*β*	Boot s.e.	BootLLCI	BootULCI	Summary	*β*	Boot s.e.	BootLLCI	BootULCI	Summary	*β*	Boot s.e.	BootLLCI	BootULCI	Summary
Low CRP	3.5	3.92	0.89	0.374	*R*^2^ = 0.30	−1.85	2.22	−6.72	2.05	*R*^2^ = 0.18	−0.93	1.21	−3.81	1.14	*R*^2^ = 0.51
High CRP	−9.76	4.52	−2.16	0.034	*F*(5,83) = 7.19, P < 0.001	5.15	2.57	0.6	10.63	*F*(4,84) = 4.76, P = 0.002	2.61	1.43	0.22	5.84	*F*(5,83) = 17.03, P < 0.001

IT, involuntary treatment; ISMIS, internalised stigma; SEPRS, self– efficacy; QPR, recovery; LLCI, lower limit confidence interval; UCIL, upper limit confidence interval; CRP, contact with recovered peers.

a. Bootstrap sample size, 5000. IT and CRP were mean-centred prior to analysis. In these models, only the effect of involuntary treatment on internalised stigma is moderated by contact with recovered peers.

### Model 2: conditional indirect effect, self-efficacy for personal recovery

As predicted, conditional internalised stigma mediated the relationship between involuntary treatment and self-efficacy (index score  7.00 (s.e. = 3.57), 95% CI 0.78–14.85). Further analysis showed that this mediation effect was significant when participants had high contact with recovered peers (*β* = 5.15 (s.e. = 2.57), 95% CI 0.60–10.63, Table 3) but not when participants had experienced low contact. Pairwise contrasts between these conditional indirect effects revealed a significant difference across the two comparison groups; compared with those with low contact, self-efficacy scores were 7.00 points higher for those with high contact with recovered peers in this model. The unconditional effect of involuntary treatment on self-efficacy was not significant.

### Model 3: conditional indirect effect, recovery

As hypothesised, the relationship between involuntary treatment experience and recovery was mediated by conditional internalised stigma and self-efficacy in sequence (index score  3.54 (s.e. = 2.03), 95% CI 0.39–8.20). Further analysis showed this was significant when participants had high contact with recovered peers (*β* = 2.61 (s.e. = 1.43), 95% CI 0.22–5.84, Table 3) but not when participants had experienced low contact. Pairwise contrasts between these conditional indirect effects revealed a significant difference across the two groups; compared with those with low contact, recovery scores were 3.54 points higher for those with high contact with recovered peers in this model. The conditional indirect effect of involuntary treatment experience on recovery through internalised stigma alone was not significant, nor was there a significant unconditional effect of involuntary treatment on recovery via self-efficacy. The unconditional effect of involuntary treatment experience on recovery was non-significant.

## Discussion

This project was grounded in an objective to advance the study of patient experiences in mental healthcare, a nascent field challenged by a limited understanding of the interrelationships between important constructs. We used statistical modelling to examine the interactive relationship between involuntary treatment and contact with recovered peers. In doing so, we have taken an initial step in addressing conceptual and measurement challenges relating to these important ideas. To the authors' knowledge, this is the first study to quantitatively examine the interactive relationship between contact with recovered peers and involuntary treatment experience, and their impact on the self-evaluative processes of stigma and efficacy known to influence recovery.

We proposed that the detrimental effects of involuntary treatment on internalised stigma, self-efficacy and recovery would be buffered by contact with people in recovery from serious mental illness, because contact with people who challenge mental illness stereotypes and model recovery might counteract the stigma and discrimination inherent in involuntary treatment practices and empower individuals to self-manage their own recovery.^8^ As a preliminary test of this explanation, we examined sequential moderation and conditional process models and found significant interactive effects of involuntary treatment and contact with recovered peers on internalised stigma, which in turn mediated the effects of involuntary treatment on self-efficacy and recovery in sequence (see Fig. 1). Findings advance understanding of how involuntary treatment experiences and contact with recovered peers influence recovery.

Although the cross-sectional nature of our data precludes causal or temporal inferences, results are consistent with our conceptualised pathway, which suggests that contact with recovered peers is helpful for people who have experienced involuntary treatment because it reduces the likelihood of internalising stigma. A possible alternative explanation is that participants with higher internalised stigma may be less likely to seek out or accept peer support, especially as internalised stigma, in theory, should reflect awareness and acceptance of social stigma.^10,33^ However, this pathway was not supported in the current study. Instead, our interaction findings suggest that contact with recovered peers may help individuals who experience involuntary treatment by modelling behaviours that serve to challenge the stigma and discrimination inherent in these particular service experiences.^34^

Findings add quantitative support to the extensive qualitative literature of patient reports that contact with recovered peers counters disempowering service experiences and facilitates recovery.^7,8,14^ These findings also align with the public stigma literature, whereby contact with people with lived experience of mental illness who disconfirm prevailing mental illness stereotypes is considered the most effective mechanism in combatting public prejudice and discrimination, resulting in reduced social distancing and greater empathic connection with people from the stigmatised group.^35^

Additionally, our findings showed that conditional internalised stigma mediated the relationship between involuntary treatment and recovery-specific self-efficacy, which in turn mediated the relationship between conditional internalised stigma and recovery. Importantly, the sequential nature of our conceptualised pathway was integral to understanding these relationships in the current study. Internalised stigma and self-efficacy did not independently mediate the conditional and/or unconditional relationship between involuntary treatment and recovery. As with previous studies, these findings suggest that stigmatising self-evaluations are detrimental to recovery in part because of their detrimental influence on self-percepts that personal recovery is possible.^15,17,18^ That these mechanisms of self-evaluation are conditional on contact with recovered peers aligns with self-efficacy theory, whereby social support, encouragement and vicarious learning through observation of successful peers modelling desired behaviours can enhance one's confidence that they too possess the capabilities to master comparable activities.^16^

Other theoretical frameworks also support the role of peers in countering the impact of being a psychiatric patient and promoting recovery.^34,36^ Social comparison theory posits that contact with recovered peers may counter the stigmatising experiences recognised as a significant barrier to recovery by facilitating upward comparisons with others ‘doing better’, who inspire hope or challenge stereotypes. The stress and coping perspective suggests that emotional and information resources needed to engage in adaptive coping and problem-solving may be provided through contact with recovered peers. Experiential knowledge has been identified as a further psychosocial process underpinning peer-provided services, promoting choice and self-determination rather than passivity commonly engendered through engagement with hierarchical service structures.

Although limitations of our cross-sectional design should be borne in mind, our findings suggest a potential direction for interventions designed to reduce the harmful effects of involuntary treatment experiences. Involuntary treatment is often viewed as a necessary intervention to manage or curtail symptoms or perceived dangerousness;^37^ however, our findings suggest that receiving treatment involuntarily may be associated with cognitive and emotional self-evaluations that could impede recovery. Creating opportunities for contact with people with lived experience of illness and recovery may provide a tangible clinical intervention for counteracting disempowering service experiences.

### Limitations, strengths and future directions

Limitations that need to be considered when interpreting these findings include the following. First, given this field is challenged by a lack of measurement and conceptual clarity pertaining to patient experiences, we were not able to use a well-established scale with known psychometric properties to measure extent of prior contact with recovered peers. Certainly, the considerable challenges operationalising peer contact are documented in the literature.^38^ Regardless, honouring patients' subjective reflections of extent and quality of contact remains important. In the present study we attempted to develop a valid and reliable measure through extensive codesign and usability monitoring of having had contact with recovered peers. Further validation of this measure is required. Additionally, future research will benefit from more specific analyses focusing on different types of contact with peers instead of using our naturalistic contact with recovered peers score. It should also be noted that contact with recovered peers has been reduced to a single variable. Different types of peer contact (such as peer support, mutual self-help, peer-led education recovery programmes) may be differently associated with recovery-inconsistent service experiences and self-evaluations relevant to recovery.

Similarly, measurement of the involuntary treatment construct was blunt. Future research, motivated by our findings here, could attempt to corroborate self-report data with hospital records. Exploring possible differences in self-evaluation mechanisms and influence on recovery according to different types of involuntary treatment (for example involuntary admission to hospital, restrictive practices, community treatment orders) and other service experiences not aligned to recovery goals (for example pressure to take medication, disempowering interactions with healthcare providers) is also needed, given that stigma, discrimination and disempowerment may be experienced differently in different treatment contexts.

Second, due to the aforementioned limitations of the cross-sectional design, a larger longitudinal study examining the impact of contact with recovered peers on the external services and intrapersonal processes associated with recovery is needed. Third, there are numerous factors that may influence internalised stigma and self-efficacy for personal recovery that were not measured or included in our analysis.

Our study also has a number of strengths. Foremost, we advance a scientific agenda by generating many more questions than we answer about patient experiences in mental health services. Historically, research has confined examination of the role of peers to formal peer-support networks or educational programmes. However, this study has captured subjective determinations of others' recovery states and honoured the potential influence of contact with people in recovery through any and all mediums. This is especially important given arguments recently advanced in the medical humanities literature that recovery narratives in mental health policy and mainstream services represent only a narrow subset of the possible experiences of recovery.^39^ Future research, informed by a broader literature (anthropology, philosophy, cultural theory, sociology), can usefully examine the subjective, objective, facilitated and spontaneous facets of this construct, and the conditions in which recovery is facilitated or impeded by peer contact (i.e. contact between individuals in different phases of wellness).

Further, to our knowledge, this is the first study to use a self-efficacy measure specific to personal recovery domains, which extends previous research that has traditionally examined self-efficacy from a more generalised perspective. According to Bandura's self-efficacy theory,^16^ confidence in one's ability to perform a particular outcome is task specific. Generalised self-efficacy measures in models pertaining to personal recovery are unlikely to adequately capture the nuance of personal recovery, given personal recovery is process-driven rather than outcome focused (see supplementary File 1 for further information). We also capitalise on the statistical advantages of using sophisticated modelling to more completely answer questions about how contact with recovered peers and involuntary treatment influence recovery. We hope this will pave the way for future research evaluating the consequences of psychiatric service experiences and the inclusion of recovered peers alongside psychiatric service delivery.

In sum, this study examined hypotheses about the moderating and mediating mechanisms by which contact with recovered peers and involuntary treatment relates to recovery from serious mental illness. We found support for our moderation and conditional process models, which incorporated moderation by contact with recovered peers and mediation by internalised stigma and self-efficacy in sequence. Although study methods limit causative conclusions, findings tentatively suggest that the value often attributed to peer support in promoting recovery may be partly related to it cushioning unintended harmful effects of involuntary treatment on processes pertinent to recovery. A model for further examination is that greater contact may protect against the internalisation of mental illness stigma, which in turn may sustain confidence, agency and ultimately personal fulfilment in the domains relevant to personal recovery.

## Supplementary material

For supplementary material accompanying this paper visit http://dx.doi.org/10.1192/bjo.2019.72. click here to view supplementary material
