# Supplementary material for: Contact with recovered peers: buffering disempowering service experiences and promoting personal recovery in serious mental illness
Source: BJPsych Open. 2019 Nov 8;5(6):e98. doi: 10.1192/bjo.2019.72 (PMC6854358; doi:10.1192/bjo.2019.72)
Supplement: Supplementary file 1 [file S2056472419000723sup001.docx]

**Supplementary File 1 Extended Introduction**

**Introduction**

Recent decades have seen significant changes in mental health service delivery, with the *recovery paradigm* progressively instantiated in policies and guidelines, particularly in English-speaking countries of the Global North (1-3). Mental health services for people diagnosed with serious and persisting mental health problems increasingly prioritise *personal recovery*, which has been defined as “a deeply personal, unique process of changing one's attitudes, values, feelings, goals, skills and/or roles. It is a way of living a satisfying, hopeful, and contributing life even with limitations caused by the illness” (Anthony, 1993, p.527). From a research viewpoint, there has been burgeoning interest in systems, services, and treatment regimens for factors that may facilitate or impede personal recovery (5-7). Two findings are particularly relevant to the present project.

*Involuntary treatment experience*

Personal recovery may be important not solely in terms of adjusting to direct limitations arising from illness, but also those associated with treatment. Particularly notable in this respect are that persons may experience episodes of involuntary treatment, such as forced treatment in hospital or mandated community treatment. A number of studies have found that involuntary treatment can be detrimental to the integrity of the self and impede agency and autonomy, as well as reinforcing prevailing, negative mental illness stereotypes (8-11). These findings inform questions about clinical efficacy and social benefit (12-15) and contribute to the sustained controversy regarding the ethics of involuntary treatment (16, 17). Still, involuntary treatment remains a common intervention in many countries (18-21).

*Mental illness internalised stigma*

Advocates for mental health law reform assert that involuntary treatment practices are inherently stigmatising and discriminatory (20, 22), and serve to reinforce the prevailing negative stereotypes about mental illness (i.e., that persons with mental illness are dangerous, incompetent, and unable to care for themselves) (23, 24). Internalised stigma, the process of endorsing, internalising, and applying mental illness stereotypes to oneself (25-28), may therefore be important in understanding the interactive effect of contact with recovered peers and involuntary treatment on recovery. Narrative accounts of service-user experiences provide support for this assertion, with reported consequences of involuntary treatment including feeling devalued, stigmatised, and dehumanised, and experiencing impaired self-esteem, agency, and autonomy (8, 9). Involuntary treatment experiences and perceptions of coercion in community treatment have been linked to harmful stigma processes, including perceived devaluation and discrimination (19), which in turn have been associated with internalised stigma (27). In a separate study of 186 individuals with serious mental illness and a history of recent involuntary hospitalisation, stigma stress, shame, and self-contempt independently predicted internalised stigma and decreased empowerment, after controlling for symptoms, diagnoses and sociodemographic variables (29).

*Contact with recovered peers*

On the other side of the coin, there is evidence that one clinically-important facilitator of recovery may be contact with peers further along the recovery pathway, or contact with recovered peers (8, 30, 31). In this context, identification as a peer is derived from matching the stigmatised minority status of people with mental illness and having shared mental health-related experiences (32). Unlike involuntary treatment, reported benefits of contact with recovered peers include countering the impact of stigma and discrimination by challenging mental illness stereotypes and offering service users a “road map for how to navigate their recovery journeys” through vicarious experiential learning (8, 9, 33). Randomised controlled trials evaluating peer-led services for people with severe mental illness have demonstrated that peer support was associated with positive effects on clinical, subjective and social outcomes, including hope, control, agency and empowerment (34-37). Extensive qualitative investigation with mental health service users has further highlighted the integral role of peers in transforming illness identities, engendering hope and belonging, and modelling self-management of one’s own recovery (9, 34, 38, 39). Quantitative investigation of whether contact with recovered peers buffers the disempowerment experienced through involuntary treatment experiences that contributes to internalised stigma is therefore an important next step.

*Self-efficacy for personal recovery*

Self-efficacy, a view of oneself as capable and agentic (40), represents a further self-evaluative mechanism that may be important for explaining how involuntary treatment, contact with recovered peers and internalised stigma impact recovery. Both internalised stigma and self-efficacy are robust predictors of personal recovery (27, 41-44), and have been previously identified as possible mechanisms through which contact and disempowering service experiences may influence recovery (9, 10). Multiple studies have shown that internalising stigma acts as a barrier to recovery by undermining self-efficacy, with lower levels of internalised stigma predicting greater self-efficacy, empowerment and recovery (27, 45, 46). Further, researchers have argued that involuntary treatment approaches contravene the core values of recovery-oriented care, including shared decision-making, self-determination, and promoting self-agency and autonomy (47), which may serve to undermine self-efficacy.

These findings suggest that disempowering service experiences (i.e., involuntary treatment) and contact with recovered peers may interact to influence recovery, and further, that this relationship may be mediated by internalised stigma and self-efficacy. To date, no systematic exploration of these relationships has been conducted. Consequently, the field is challenged by a lack of measurement and conceptual clarity. Additionally, investigations of the relationships between internalised stigma, self-efficacy and recovery have previously relied on general measures of self-efficacy or singular aspects of recovery, including social connectedness, empowerment, and self-management (48-50). A personal recovery-specific measure of self-efficacy is better aligned with Bandura’s self-efficacy theory, which specifies that self-evaluative beliefs are specific to tasks or domains (51, 52). In the context of serious mental illness, specific domains pertaining to personal recovery and self-management can be summarised by the CHIME acronym and include: developing and maintaining supportive relationships (connectedness); feeling motivated and believing in one’s ability to enact change (hope); developing positive personal and social identities beyond a stigmatised ‘passive patient’ role (identity); living a subjectively meaningful and purposeful life (meaning); and accessing agency and autonomy in recovering one’s life (empowerment) (53).

*The present study*

In order to advance the scientific study of service user experiences in mental health services, the aim of the present study was to systematically examine the interactive relationship between involuntary treatment experience and contact with recovered peers, and the intrapersonal mediating processes that may help to explain their impact on personal recovery. We hypothesised that: (H1) contact with recovered peers moderates the effect of involuntary treatment on internalised stigma; (H2) conditional internalised stigma (i.e., moderated by contact with recovered peers) mediates the indirect effect of involuntary treatment on recovery-specific self-efficacy; and (H3) self-efficacy mediates the conditional indirect effect of internalised stigma on recovery. The full complexity of this moderated multiple-mediation model was tested using conditional process analysis (i.e., ordinary least squares regression-based path analysis), and our predictions are captured in the conceptual models of Figure 1. In the hypothesised (conditional) models, only the effect of involuntary treatment on internalised stigma is moderated by contact with recovered peers.

**Additional References**

1. Commonwealth of Australia. Fourth national mental health plan. In: Australia Co, editor. Barton, ACT: Commonwealth of Australia; 2009.

2. UK Department of Health. No health without mental health. London2011.

3. US Dept Health and Human Services. Achieving the promise. Rockville, Maryland: Commission on mental health; 2003.

4. Anthony WA. Recovery from mental illness: the guiding vision of the mental health service system in the 1990s. Psychosocial rehabilitation journal. 1993;16(4):11.

5. Australian Health Ministers' Advisory Council. A national framework for recovery-oriented mental health services: Guide for practitioners and providers. Canberra, Australia: Commonwealth of Australia; 2013.

6. Bellack AS. Scientific and consumer models of recovery in schizophrenia: Concordance, contrasts, and implications. Schizophrenia Bulletin. 2006;32(3):432-42.

7. Morgan VA, Waterreus A, Carr V, Castle D, Cohen M, Harvey C, et al. Responding to challenges for people with psychotic illness: Updated evidence from the Survey of High Impact Psychosis. Australian & New Zealand Journal of Psychiatry. 2017;51(2):124-40.

8. Mancini MA, Hardiman ER, Lawson HA. Making sense of it all: Consumer providers' theories about factors facilitating and impeding recovery from psychiatric disabilities. Psychiatric Rehabilitation Journal. 2005;29(1):48-55.

9. Hughes R, Hayward M, Finlay WML. Patients’ perceptions of the impact of involuntary inpatient care on self, relationships and recovery. Journal of Mental Health. 2009;18(2):152-60.

10. Tew J, Ramon S, Slade M, Bird V, Melton J, Le Boutillier C. Social factors and recovery from mental health difficulties: a review of the evidence. British journal of social work. 2011:bcr076.

11. Davidson L, Strauss JS. Sense of self in recovery from severe mental illness. British Journal of medical psychology. 1992;65(2):131-45.

12. Priebe S, Katsakou C, Yeeles K, Amos T, Morriss R, Wang D, et al. Predictors of clinical and social outcomes following involuntary hospital admission: a prospective observational study. European archives of psychiatry and clinical neuroscience. 2011;261(5):377-86.

13. Newton-Howes G, Mullen R. Coercion in psychiatric care: systematic review of correlates and themes. Psychiatric Services. 2011;62(5):465-70.

14. Barnett P, Matthews H, Lloyd-Evans B, Mackay E, Pilling S, Johnson S. Compulsory community treatment to reduce readmission to hospital and increase engagement with community care in people with mental illness: a systematic review and meta-analysis. The Lancet Psychiatry. 2018.

15. Kisely SR, Campbell LA, O'Reilly R. Compulsory community and involuntary outpatient treatment for people with severe mental disorders. Cochrane database of systematic reviews. 2017(3).

16. Brophy L, McDermott F. What's driving involuntary treatment in the community? The social, policy, legal and ethical context. Australasian Psychiatry. 2003;11(sup1):S84-S8.

17. Kallert TW, Mezzich JE, Monahan J. Coercive treatment in psychiatry: clinical, legal and ethical aspects: John Wiley & Sons; 2011.

18. Katsakou C, Priebe S. Patient's experiences of involuntary hospital admission and treatment: a review of qualitative studies. Epidemiology and Psychiatric Sciences. 2007;16(2):172-8.

19. Link B, Castille DM, Stuber J. Stigma and coercion in the context of outpatient treatment for people with mental illnesses. Social science & medicine. 2008;67(3):409-19.

20. Bates L, Stickley T. Confronting Goffman: how can mental health nurses effectively challenge stigma? A critical review of the literature. Journal of psychiatric and mental health nursing. 2013;20(7):569-75.

21. Australian Institute of Health and Welfare. Mental health services in Australia: Restrictive practices. Australian Government; 2018.

22. Sartorius N. Iatrogenic stigma of mental illness: begins with behaviour and attitudes of medical professionals, especially psychiatrists. British Medical Journal Publishing Group; 2002.

23. Link BG, Struening EL, Neese-Todd S, Asmussen S, Phelan JC. Stigma as a barrier to recovery: The consequences of stigma for the self-esteem of people with mental illnesses. Psychiatric Services. 2001;52(12):1621-6.

24. Corrigan PW, Watson AC. The Paradox of Self-Stigma and Mental Illness. Clinical Psychology: Science and Practice. 2002;9(1):35-53.

25. Link BG, Phelan JC. Conceptualizing Stigma. Annual Review of Sociology. 2001;27:363-85.

26. Link BG, Cullen FT, Struening E, Shrout PE, Dohrenwend BP. A modified labeling theory approach to mental disorders: an empirical assessment. American Sociological Review. 1989:400-23.

27. Corrigan PW, Larson JE, Ruesch N. Self‐stigma and the “why try” effect: impact on life goals and evidence‐based practices. World psychiatry. 2009;8(2):75-81.

28. Thomas N, McLeod B, Jones N, Abbott J. Developing internet interventions to target the individual impact of stigma in health conditions. Internet Interventions. 2015;2.

29. Rüsch N, Müller M, Lay B, Corrigan PW, Zahn R, Schönenberger T, et al. Emotional reactions to involuntary psychiatric hospitalization and stigma-related stress among people with mental illness. European Archives of Psychiatry and Clinical Neuroscience. 2014;264(1):35-43.

30. Castelein S, Bruggeman R, Van Busschbach JT, Van Der Gaag M, Stant A, Knegtering H, et al. The effectiveness of peer support groups in psychosis: a randomized controlled trial. Acta Psychiatrica Scandinavica. 2008;118(1):64-72.

31. Verhaeghe M, Bracke P, Bruynooghe K. Stigmatization and self-esteem of persons in recovery from mental illness: The role of peer support. International Journal of Social Psychiatry. 2008;54(3):206-18.

32. Simoni JM, Franks JC, Lehavot K, Yard SS. Peer interventions to promote health: Conceptual considerations. American Journal of Orthopsychiatry. 2011;81(3):351.

33. Mancini MA. The role of self-efficacy in recovery from serious psychiatric disabilities: A qualitative study with fifteen psychiatric survivors. Qualitative Social Work. 2007;6(1):49-74.

34. Cook JA, Copeland ME, Corey L, Buffington E, Jonikas JA, Curtis LC, et al. Developing the evidence base for peer-led services: Changes among participants following Wellness Recovery Action Planning (WRAP) education in two statewide initiatives. Psychiatric Rehabilitation Journal. 2010;34(2):113-20.

35. Cook JA, Copeland ME, Jonikas JA, Hamilton MM, Razzano LA, Grey DD, et al. Results of a randomized controlled trial of mental illness self-management using wellness recovery action planning. Schizophrenia Bulletin. 2012;38(4):881-91.

36. Lloyd-Evans B, Mayo-Wilson E, Harrison B, Istead H, Brown E, Pilling S, et al. A systematic review and meta-analysis of randomised controlled trials of peer support for people with severe mental illness. BMC psychiatry. 2014;14(1):39.

37. Slade M, Amering M, Farkas M, Hamilton B, O'Hagan M, Panther G, et al. Uses and abuses of recovery: Implementing recovery-oriented practices in mental health systems. World Psychiatry. 2014;13(1):12-20.

38. Mead S, Copeland ME. What recovery means to us: Consumers' perspectives. Community Mental Health Journal. 2000;36(3):315-28.

39. Davidson L, Schmutte T, Dinzeo T, Andres-Hyman R. Remission and recovery in schizophrenia: Practitioner and patient perspectives. Schizophr Bull. 2008;34.

40. Bandura A. Self-efficacy: toward a unifying theory of behavioral change. Psychological review. 1977;84(2):191.

41. Whitley R, Drake RE. Recovery: a dimensional approach. Psychiatr Serv. 2010;61.

42. Law H, Morrison AP. Recovery in psychosis: A Delphi study with experts by experience. Schizophr Bull. 2014;40.

43. Chronister J, Chou CC, Liao HY. The role of stigma coping and social support in mediating the effect of societal stigma on internalized stigma, mental health recovery, and quality of life among people with serious mental illness. Journal of Community Psychology. 2013;41(5):582-600.

44. Corrigan PW, Watson AC, Barr L. The self-stigma of mental illness: implications for self-esteem and self-efficacy. Journal of Social and Clinical Psychology. 2006;25(8):875-84.

45. Kleim B, Vauth R, Adam G, Stieglitz R-D, Hayward P, Corrigan P. Perceived stigma predicts low self-efficacy and poor coping in schizophrenia. Journal of Mental Health. 2008;17(5):482-91.

46. Vauth R, Kleim B, Wirtz M, Corrigan PW. Self-efficacy and empowerment as outcomes of self-stigmatizing and coping in schizophrenia. Psychiatry research. 2007;150(1):71-80.

47. Farkas M, Gagne C, Anthony W, Chamberlin J. Implementing recovery oriented evidence based programs: Identifying the critical dimensions. Community mental health journal. 2005;41(2):141-58.

48. Wright SL, Wright DA, Jenkins-Guarnieri MA. Development of the social efficacy and social outcome expectations scale. Measurement and Evaluation in Counseling and Development. 2013;46(3):218-31.

49. Carpinello SE, Knight EL, Markowitz FE, Pease EA. The development of the Mental Health Confidence Scale: A measure of self-efficacy in individuals diagnosed with mental disorders. Psychiatric Rehabilitation Journal. 2000;23(3):236.

50. Clarke J, Proudfoot J, Birch M-R, Whitton AE, Parker G, Manicavasagar V, et al. Effects of mental health self-efficacy on outcomes of a mobile phone and web intervention for mild-to-moderate depression, anxiety and stress: secondary analysis of a randomised controlled trial. BMC Psychiatry. 2014;14(1):272.

51. Villagonzalo K-A, Leitan N, Farhall J, Foley F, McLeod B, Thomas N. Development and validation of a scale for self-efficacy for personal recovery in persisting mental illness. Psychiatry Research. 2018;269:354-60.

52. Bandura A. Social foundations of thought and action. Englewood Cliffs, NJ. 1986;1986.

53. Leamy M, Bird V, Le Boutillier C, Williams J, Slade M. Conceptual framework for personal recovery in mental health: systematic review and narrative synthesis. The British Journal of Psychiatry. 2011;199(6):445-52.
